# Hypotensive effect of *Gentiana floribunda* is mediated through Ca^++^ antagonism pathway

**DOI:** 10.1186/1472-6882-12-121

**Published:** 2012-08-11

**Authors:** Arif-ullah Khan, Mohamad Rais Mustafa, Asif Ullah Khan, Dharmani Devi Murugan

**Affiliations:** 1 Centre for Natural Products Research and Drug Discovery, Department of Pharmacology, Faculty of Medicine, University of Malaya, Kuala Lumpur 50603, Malaysia; 2 Department of Pharmacy, Kohat University of Science and Technology, Kohat 26000, KPK, Pakistan; 3 Institute of Basic Medical Sciences, Khyber Medical University, Peshawar, Pakistan

## Abstract

**Background:**

*Gentiana floribunda* was investigated for the possible hypotensive and vasodilator activities in an attempt to rationalize its traditional use in hypertension.

**Methods:**

The crude extract of *Gentiana floribunda* (Gf.Cr) was studied in anaesthetized rats and isolated thoracic aorta tissues.

**Results:**

Gf.Cr which tested positive for presence of flavonoids, saponins, sterols, tannins and terpenes caused dose-dependent (3.0-100 mg/kg) fall in arterial blood pressure (BP) of rats under anaesthesia. In rat aortic ring preparations denuded of endothelium, Gf.Cr at concentration range of 1.0-10 mg/mL relaxed high K^+^ (80 mM) and phenylephrine (PE, 1 μM)-induced contractions and shifted Ca^++^ dose–response curves to right, similar to that caused by verapamil. It also suppressed PE (1 μM) control peak responses at 0.3-1.0 mg/mL, obtained in Ca^++^-free medium, as exhibited by verapamil. Pre-treatment of tissues with Gf.Cr produced rightward non-parallel shift of PE-curves with decline of maximum contractile response. The vasodilator effect of Gf.Cr was endothelial-independent, as it was not blocked by N_ω_-nitro-L-arginine methyl ester hydrochloride, atropine and indomethacin in endothelium-intact aortic tissues.

**Conclusions:**

These data indicate that BP-lowering action of *Gentiana floribunda* occurred via Ca^++^ antagonism (inhibition of Ca^++^ ingress and release from intracellular stores), which provides pharmacological basis to justify its effectiveness in hypertension.

## Background

Hypertension is one of the most common and rapidly spreading cardiovascular diseases, which is a major cause of morbidity and mortality in mankind and an important risk factor for the development of other cardiovascular diseases [1]. The pathophysiologic basis for over 90 percent cases of disease remains unexplained, and therefore, the condition is called primary or essential hypertension. The heritability of essential hypertension is estimated to be about 30%. The remaining 5–10 percent may be due to a number of known causes, including renal artery stenosis, aortic coarctation, cushing’s syndrome and pheochromocytoma, called secondary hypertension [2]. Hypertension is a serious affliction that is often asymptomatic. Over time, it leads to a variety of health problems including premature sickness, stroke, congestive heart failure, myocardial infarction, peripheral vascular disease, retinopathy, dementia, renal dysfunction, cerebro-vascular damage, disability and death in the adult population [3]. The hypertension-related morbidity and mortality are directly related to the level of blood pressure (BP). The incidence of morbidity and mortality significantly decreases when hypertension is diagnosed early and is properly treated, whereas untreated hypertension is known as the ‘silent killer’. The risk of developing a cardiovascular complication is higher when the individual combines hypertension with other risk factors, such as hypercholesterolemia, diabetes and a family history of cardiovascular diseases [4]. The conventional therapy for the hypertension is not always safe, efficacious and is beyond the access and/or affordability of large proportion of world population, who looks for alternative therapeutic measures [5]. Phytotherapy is the most popular alternative remedy and a number of traditional systems of medicine are heavily based on the use of herbs as medicine. At the same time, there is a global revival of interest in the use of botanicals and physicians of the modern medicine are now beginning to accept the traditional remedies following scientificant validation [6]. Previously, few species of the genus “*Gentiana”* were evaluated for cardiovascular inhibitory activities. For example, *Gentiana olivieri* and *Gentiana kokiana* are reported to exhibit hypotensive and vasodilatory actions respectively [7,8]. *Gentiana floribunda* Don. (Gentianaceae) is an erect annual or perennial herb, native of Himalaya region. The plant is used in a traditional medical system for the treatment of variety of health ailments, such as hypertension, gastrointestinal motility, congestive respiratory, hepato-biliary and neurological disorders [9,10]. Despite the fact that *Gentiana floribunda* has been used medicinally, it has not been widely studied scientifically. In view of our project aimed at the ethnopharmacological evaluation of medicinal plants for cardiovascular effects, the present research was carried out to explore the possible mechanism of action involved in the hypotensive potential of *Gentiana floribunda*. We report here that it exhibits BP-lowering and endothelium-independent vasodilatory effects, mediated through dual inhibition of Ca^++^ influx across membranous voltage-sensitive and receptor-operated Ca^++^ channels as well as its release from intracellular sarcoplasmic reticulum stores. This study provides a sound mechanistic background for the medicinal application of *Gentiana floribunda* in hypertension.

## Methods

### Plant material and extraction

*Gentiana floribunda* whole plant material was collected from Kurram valley, Pakistan in 2009 and identified with help of botanical authority at Kohat University of Science and Technology, Kohat, Pakistan and voucher specimen was deposited at Herbarium of the same University. The plant material was cleaned, shade dried and coarsely ground. The powdered material (1.5 kg) was soaked in 70% aqueous-methanol solution in a large container for 3 days with occasional shaking. It was filtered through a muslin cloth and then through filter paper [11]. This procedure was repeated twice more and the combined filtrate was evaporated on a rotary evaporator, under reduced pressure to a thick semi-solid mass of dark brown color i.e. the crude extract of *Gentiana floribunda* (Gf.Cr, 250 g), yielding 16.7%. Gf.Cr was solubilized both in saline and distilled water.

### Chemicals

The following reference chemicals were obtained from the sources specified: acetylcholine chloride (ACh), atropine, indomethacin, N_ω_-nitro-L-arginine methyl ester hydrochloride (L-NAME), norepinephrine hydrochloride (NE), phentolamine hydrochloride, phenylephrine hydrochloride (PE), urethane and verapamil hydrochloride (Sigma Chemical Company, St. Louis, MO, USA). The following chemicals were used to make physiological salt solutions: potassium chloride (Sigma Chemical Company, St. Louis, MO, USA), calcium chloride, glucose, magnesium sulphate, potassium dihydrogen phosphate, sodium bicarbonate, sodium chloride (Merck, Darmstadt, Germany) and ethylenediaminetetra-acetic acid (EDTA) from BDH Laboratory Supplies, Poole, England. The chemicals used in phytochemical analysis include: acetic anhydride, aluminum chloride, ammonium hydroxide, ferric chloride (Sigma Chemical Co, St Louis, MO, USA), benzene, chloroform, hydrochloric acid and petroleum ether (BDH Laboratory supplies, Poole, England). All chemicals used were of the highest analytical grade available.

### Phytochemical screening

Preliminary phytochemical screening of the extract was carried out for the presence of anthraquinones, coumarins, flavonoids, saponins, sterols, tannins and terpenes in accordance to the reported procedures [12]. Presence of saponins was detected based on the appearance of froth upon vigorous shaking of diluted samples. The observation of yellow florescence under ultraviolet light on examination of filter paper previously exposed to the vapors from boiling plant material indicated the presence of coumarins. For the detection of sterols and terpenes, plant material was treated with petroleum ether and subsequently extracted with CHCl_3_. The gradual appearance of green to pink (for sterols) and pink to purple color (for terpenes) was then noted after treatment of CHCl_3_ layer with acetic anhydride and concentrated H_2_SO_4_ in succession. Plant material was detected as positive for flavonoids when it gave yellow color with AlCl_3_ reagent and for tannins, when green or black color was produced with aqueous FeCl_3_. Lastly, for detecting anthraquinones, the extract was dissolved in 1% HCl, then in benzene and later if extract showed pink, violet or red color with NH_4_OH, that indicate the presence of anthraquinones.

### Animals

Male Sprague–Dawley rats (240–260 g) of local breed were housed at animal house of the Department of Pharmacology, University of Malaya, under controlled environment (23-25 °C). Animals were given tap water *ad libitum* and standard diet. Experiments performed complied with rulings of the Institute of Laboratory Animal Resources, Commission on Life Sciences, National Research Council [13], approved by University of Malaya Animal Experimentation Ethics Committee.

### Measurement of blood pressure

These experiments were performed according to method described previously [14]. Rats were anaesthetized with urethane (1.2-1.5 g/kg, i.p.). Animal was fixed in supine position on a dissecting table. A small mid-tracheal incision (approx. 1 cm) was made to expose trachea, right jugular vein and left carotid artery. The trachea was cannulated with a polyethylene tubing Pe-20 to maintain the spontaneous respiration and cleaned from time to time. The right jugular vein was cannulated with polyethylene tubing Pe-50 to facilitate the intravenous administration of drugs. The left carotid artery was cannulated with similar tubing filled with heparinized saline 60 IU/mL and connected to a pressure transducer (MLT 0380/D Reusable BP- Transducer) coupled to ML 224 Quad Bridge Amplifier and PowerLab ML 4/30 data acquisition system (AD Instruments, Sydney, Australia) for BP recording. The exposed surface for cannulation was covered with a piece of gauze moistened in warm saline. Rats were injected with heparinized 0.1 mL saline (0.9% NaCl) to prevent blood clotting. Body temperature of the animal was maintained by using overhead lamp. Following 20 min period of equilibrium, rats were injected intravenously with test substance. Arterial BP was allowed to return to resting level between injections. Changes in BP were recognized as difference between the steady state values before and the peak readings after injection. Mean arterial blood pressure (MABP) was calculated as the diastolic BP plus one-third of the pulse width (systolic BP - diastolic BP). The ACh (1 μg/kg) and NE (1 μg/kg) control responses were obtained before the administration of any test material.

### Isolated rat aorta preparations

Rats were sacrificed by cervical dislocation. After abodominal opening, the thoracic aorta was dissected out, cleaned of fat and adipose tissues and cut into 3–5 mm long rings and individually mounted in 5 mL tissue bath containing Kreb’s solution composed of mM): NaCl 118.2, NaHCO_3_ 25.0, CaCl_2_ 2.5, KCl 4.7, KH_2_PO_4_ 1.3, MgSO_4_ 1.2 and glucose 11.7 (pH 7.4). The bath solution was maintained at 37 °C and continuously aerated with carbogen (95% O_2_ in 5% CO_2_). A resting tension of 1 g was applied to each tissue and an equilibrium period of 30 min was allowed before any experimentation. The tissues were then stabilized with repeated exposure (usually 3-times) to high KCl solution [15]. In experiments using endothelium-denuded tissues, endothelium lining of the aortic rings was removed mechanically by gentle rubbing with blunted forceps. Denudation of endothelium was confirmed by the absence of relaxation to ACh, 0.1-0.3 μM [16]. The test drug was tested for its ability to relax the contractions, induced with high K^+^ (80 mM) and PE, 1 μM. The ability of extract to relax K^+^ (80 mM)-induced contractions would indicate L-type voltage-operated calcium channel blocking (CCB) mode of vasodilation, while inhibition of the PE-induced contractions, would signify blockade of the Ca^++^ influx through receptor-operated calcium channels [17,18]. To confirm CCB activity, concentration-response curves (CRC_S_) of Ca^++^ were constructed [19]. For this purpose tissue was stabilized in normal Kreb’s solution and then placed in Ca^++^-free Kreb’s solution, containing EDTA (0.1 mM) for 30 min to remove calcium from the tissues. This solution was further replaced with K^+^-rich and Ca^++^-free Kreb’s solution, having the following composition (mM): KCl 50, NaCl 50.58, MgSO_4_ 3.10_._, NaHCO_3_ 23.8, KH_2_PO_4_ 1.26, glucose 11.1 and EDTA 0.1. Following an incubation period of 1 hr, control CRC_S_ of Ca^++^ were obtained. When the control CRC_S_ of Ca^++^ were found super-imposable (usually after two cycles), the tissue was pre-treated with test drug for 50–60 min for the possible CCB effect. The Ca^++^-CRC_S_ were reconstructed in presence of different concentrations of the test material. In order to determine if the extract was inhibiting Ca^++^ release from intracellular stores, the effect of increasing concentrations of extract was observed on PE (1 μM) peaks obtained in the Ca^++^-free environment (Ca^++^ omitted and EDTA (0.1 mM) added) to ensure total elimination of extracellular Ca^++^ without harmful effects on Ca^++^ inside the cell [20]. In Ca^++^-free medium, PE acts through stimulation of α_1_-adrenergic receptors. Consequent conversion of phosphatidylinositol to inositol triphosphate (IP_3_), which in turn releases Ca^++^ from the intracellular stores, brings about the contraction [21]. To assess the presence of any competitive adrenergic antagonism, cumulative curves to PE were constructed using increasing concentration of agonist. When 3-fold increase in concentration produced no further increment in response, the tissue was washed to re-establish the base-line tension (within 30–35 min). The PE-curves were then re-determined in the presence of test material. To study whether or not the vasodilator effect of test substances is endothelium-dependent, endothelium-intact aortic rings were preincubated with L-NAME (0.1 mM), atropine (1 μM) and indomethacin (1 μM) for 60 min prior to PE (1 μM)-induced contractions [22]. The endothelial integrity of aortic ring was indicated by administration of ACh (0.1 μM) on PE-induced contraction, resulting in vasorelaxation [23]. Changes in tension were recorded and analyzed isometrically, using force transducers of Multi Wire Myograph system-Model 610 M-version 2.2 (DMT A/S, Skejbyparken152, 8200 Aarhus N., Denmark) coupled to PowerLab ML 8/30 data acquisition system (AD Instruments, Sydney, Australia).

### Acute toxicity test

Mice were divided in groups of five mice each. The test was performed using increasing doses of the plant extract, given orally in 10 mL/kg volume to different groups serving as test groups. Another group of mice was administered saline (10 mL/kg, p.o.) as negative control. The mice were allowed food *ad libitum* and kept under regular observation for lethality recorded after 24 hrs [24].

### Statistical analysis

All the data is expressed as mean ± SEM and the median effective concentrations (EC_50_) values are given as geometric mean with 95% confidence intervals (CI). CRCs were analyzed by nonlinear regression (Sigmoidal dose–response curve variable slop). The statistical parameter applied was one way analysis of variance (ANOVA). Difference of *p* < 0.05 was considered statistically significant. All the graphs, calculation and statistical analyses were performed using GraphPad Prism software version 4.00 for Windows (GraphPad Software, San Diego California USA).

## Results

### Phytochemical analysis

Gf.Cr was found to contain flavonoids, saponins, sterols, tannins and terpenes, while tested negative for the rest of classes.

### Effect on blood pressure

**Figure 1:**
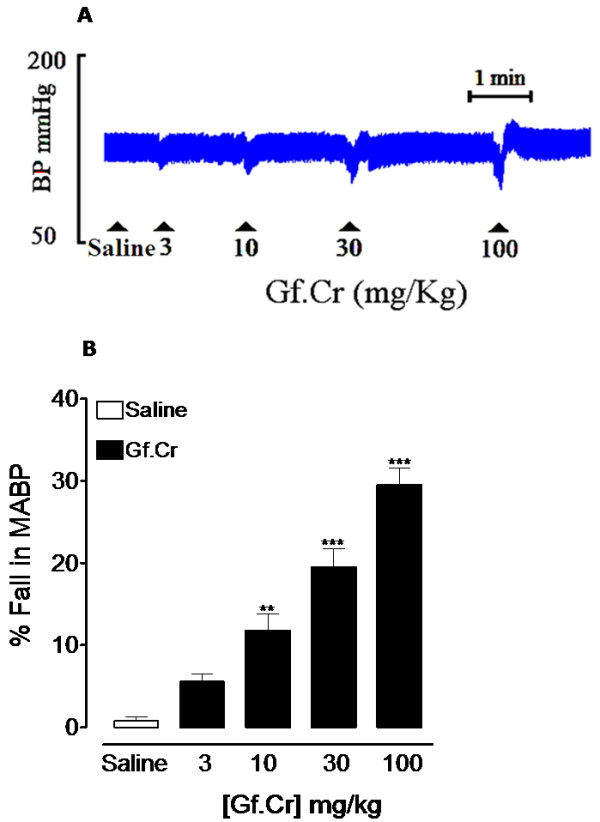
**Upper panel (A) shows a typical tracing of *Gentiana floribunda *(Gf.Cr) blood pressure (BP) lowering effect and the lower panel (B) shows a bar chart representing effect of saline (0.1 mL) and Gf.Cr on mean arterial blood pressure (MABP) in anesthetized rats.** The dose was administered after the response to the preceding one had returned to normal. Values shown represent mean ± SEM, n = 4. ^**^p < 0.01 and ^***^p < 0.001 vs. saline treatment.

Gf.Cr at the doses of 3, 10, 30 and 100 mg/kg caused a respective fall of 5.5 ± 1.0, 11.7 ± 2.0, 19.5 ± 2.2 and 29.5 ± 2.1% (n = 4) in the MABP (*p* < 0.01 and *p* < 0.001 vs. saline) of anaesthetized rats. Figure1A shows tracing from a typical experiment, whereas combined data from different experiments are presented in Figure1B.

### Effect on high K^+^ and PE-induced contractions

**Figure 2:**
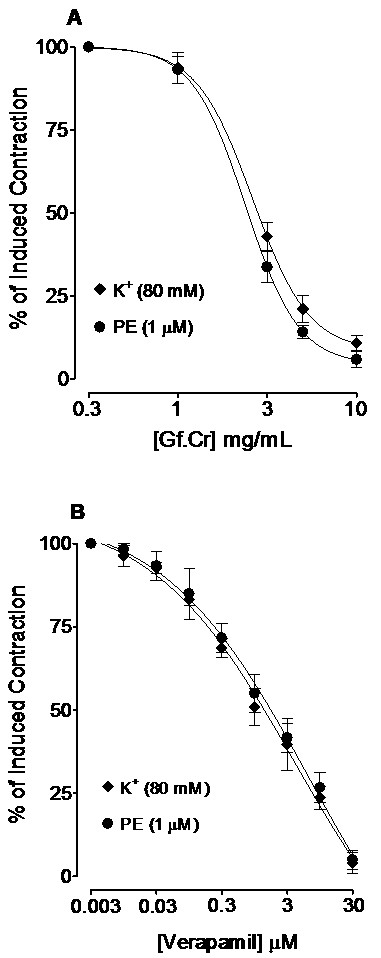
**Concentration-dependent relaxant effects of (A) crude extract of *Gentiana floribunda *(Gf.Cr) and (B) verapamil on high K**^**+**^**and phenylephrine (PE)-induced contractions in isolated rat aortic ring preparations.** Values shown are mean ± SEM, n = 4. The K^+^ and PE curves in each graph are non-significantly different (*p* > 0.05).

When tested on the resting base-line of endothelium-denuded aortic preparations, the extract was devoid of vasoconstrictor effect up to 10 mg/mL. When tested on K^+^ (80 mM) and PE (1 μM)-induced contractions, Gf.Cr produced non-specific (*p* > 0.05) vasodilator effect with respective EC_50_ values of 2.7 (2.2-3.1, n = 4) and 2.4 mg/mL (2.1-2.7, n = 4) as shown in Figure2A. Verapamil was also free of any vasoconstrictor effect and inhibited the K^+^ (80 mM) and PE (1 μM)-induced vascular contractions (*p* > 0.05) with EC_50_ values of 1.6 (0.76-3.1, n = 4) and 1.9 μM (0.84-4.9, n = 4) respectively (Figure2B).

### Effect on Ca^++^-curves

**Figure 3:**
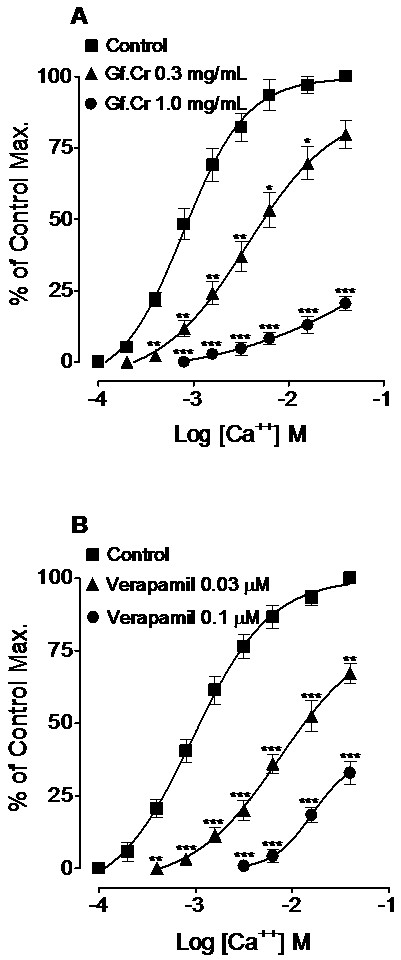
**Concentration-response curves of Ca**^**++**^**in the absence and presence of different concentrations of (A) crude extract of *Gentiana floribunda *(Gf.Cr) and (B) verapamil in isolated rat aortic ring preparations.** Values shown are mean ± SEM, n = 4. ^*^p < 0.05, ^**^p < 0.01 and ^***^p < 0.001 compared to respective concentrations values in the Ca^++^ control curves.

Gf.Cr in a concentrations-dependent manner (0.3-1.0 mg/mL, n = 4) shifted the Ca^++^-CRCs (*p* < 0.05, *p* < 0.01 and *p* < 0.001) to the right with suppression of the maximum contraction (Figure3A). This rightward shift of Ca^++^-curves was similar to the one obtained under the influence of verapamil (0.03-0.1 μM, n = 4, *p* < 0.01 and *p* < 0.001) as shown in Figure3B.

### Effect on PE-peak responses

**Figure 4:**
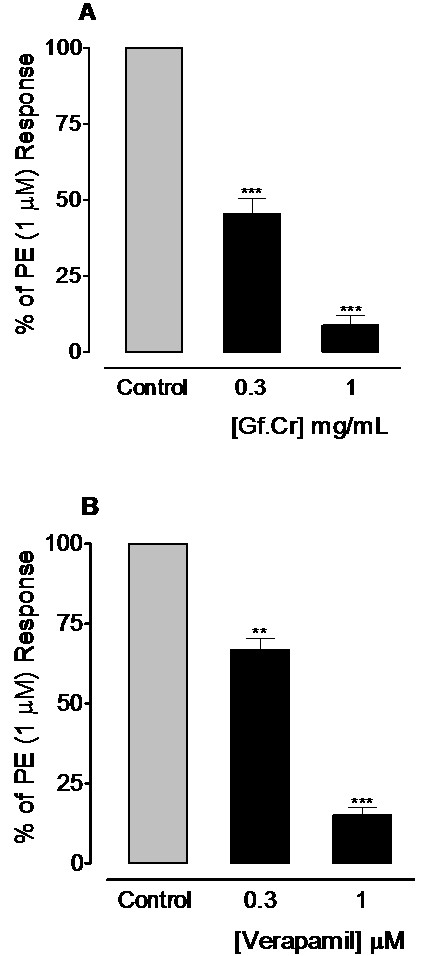
**Inhibitory effect of increasing concentrations of (A) crude extract of *Gentiana floribunda *(Gf.Cr) and (B) verapamil on control phenylephrine (PE) peak responses in Ca**^**++**^**free medium in isolated rat aortic ring preparations.** Values shown are mean ± SEM, n = 4. ^**^p < 0.01 and ^***^p < 0.001 compared to PE control peaks.

In Ca^++^-free medium, Gf.Cr at the concentrations of 0.3 and 1.0 mg/mL caused 54.5 ± 5.1 and 91.3 ± 3.2% (n = 4) suppression (*p* < 0.001) of PE (1 μM) peak responses respectively (Figure4A). This response was similar to verapamil, which inhibited the PE-peaks at 0.3 and 1.0 μM by 33.25 ± 3.5 and 85.0 ± 2.5% (n = 4) respectively (*p* < 0.01 and *p* < 0.001) as shown in Figure4B.

### Effect on PE-curves

**Figure 5:**
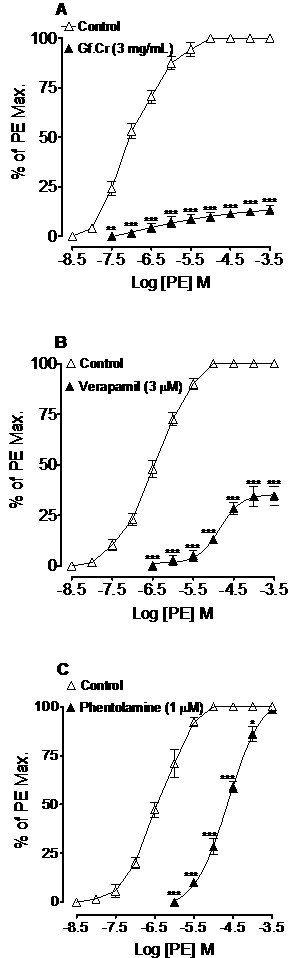
**Concentration-response curves of phenylephrine (PE) in the absence and presence of (A) crude extract of *Gentiana floribunda *(Gf.Cr), (B) verapamil and (C) phentolamine in isolated rat aortic ring preparations.** Values shown are mean ± SEM, n = 4. ^*^p < 0.05, ^**^p < 0.01 and ^***^p < 0.001 compared to respective concentrations values in the PE control curves.

PE cumulative CRCs were constructed in the absence and presence of test drugs. Gf.Cr at 3 mg/mL shifted the PE-curves to the right in non-parallel fashion with suppression (*p* < 0.001) of the agonist maximal response (Figure5A). This response was similar to that caused by verapamil at 3 μM (*p* < 0.001) as shown in Figure5B, while phentolamine (1 μM) caused rightward (*p* < 0.05 and *p* < 0.001) parallel shift without suppressing maximum contractile effect (Figure5C).

### Effect on endothelium-intact tissues

**Figure 6:**
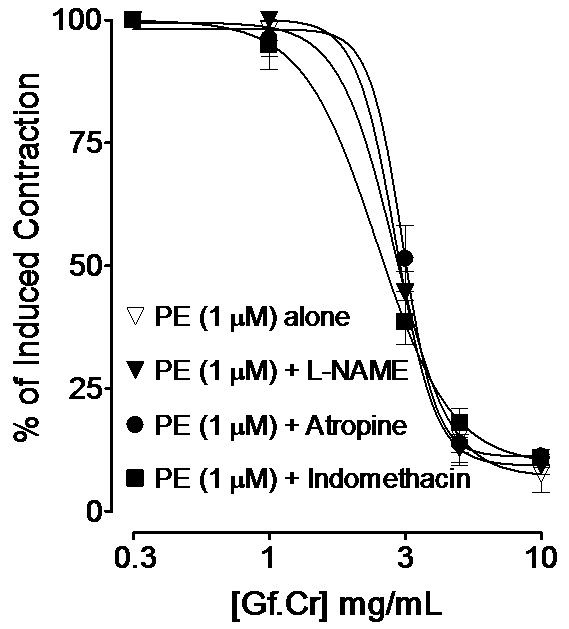
**Inhibitory effect of the crude extract of *Gentiana floribunda *(Gf.Cr) on PE (1 μM)-induced contractions in the absence and presence of N**_**ω**_**-nitro-L-arginine methyl ester hydrochloride (L-NAME, 0.1 mM), atropine (1 μM) and indomethacin (1 μM) in endothelium-intact aortic preparations from isolated rat aorta. Values shown are mean ± SEM, n = 4.** The curves are non-significantly different (*p* > 0.05).

In endothelium-intact rat aortic rings, Gf.Cr relaxed the PE (1 μM)-induced contractions (*p* > 0.05) in absence of any intervention and in presence of L-NAME (0.1 mM), atropine (1 μM) and indomethacin (1 μM) with respective EC_50_ values of 2.8 (2.5-3.3, n = 4), 2.9 (2.7-3.1, n = 4), 3.0 (2.8-3.2, n = 4) and 2.6 mg/mL (2.1-2.9, n = 4) as shown in Figure6.

### Preliminary toxicity study

The two different groups of mice were given Gf.Cr in the graded doses of 1 and 3 g/kg respectively and animals were observed for mortality after 24 hrs of drug administration. The extract did not cause any mortality up to the dose of 3 g/kg.

## Discussion

The intravenous administration of hydro-methanolic extract of *Gentiana floribunda* caused a dose-dependent fall in the arterial BP of rats, which is in line with its medicinal use in hypertension. It is customary to use isolated vascular tissue preparations to investigate the possible mode of hypotensive action, as response interference from intact reflex is obliterated [25]. To see effect on vascular resistance, Gf.Cr was studied in rat thoracic aorta, which is a prototype tissue routinely used for evaluating underlying pharmacodynamic of BP-lowering effect [26]. Rat aorta was selected to: a) evaluate effect of the extract on K^+^ and PE-induced contractions and thus to distinguish between activity at voltage-operated and receptor-operated calcium channels, b) distinguish between inhibitory effects of test drug on membrane bound Ca^++^ channels and those inside the cells and c) determine if the vasodilator effect of Gf.Cr is endothelium-independent or-dependent. Gf.Cr inhibited the PE and high K^+^-induced contractions in endothelium-denuded rings at a similar concentration range, indicating that it was acting equipotently through blockade of voltage- and receptor-operated Ca^++^ channels [27,28]. The results were similar to those obtained with verapamil, a standard Ca^++^ antagonist [26]. The CCB activity of the extract was confirmed when it shifted the Ca^++^-CRCs, constructed in Ca^++^-free medium to the right. Verapamil also caused similar rightward of Ca^++^-curves in concentration-dependent manner. Smooth muscle contraction is brought about by activation of the 1) membrane bound Ca^++^ channels which are: voltage-dependent and receptor-operated Ca^++^ channels [29], but this is not the only mechanism for contractility. Ca^++^ influx into the cell can also be guided through 2) Ca^++^ release from internal stores, like IP_3_-sensitive sarcoplasmic reticulum as well [30]. To assess the activity of extract on Ca^++^ release from intracellular stores, PE control responses were taken in absence and presence of Gf.Cr in Ca^++^-free environment. The extract in increasing concentrations suppressed the agonist peaks, similar to that caused by verapamil, indicating inhibition of Ca^++^ release from internal stores which leads to reduction of intracellular Ca^++^ content. Pre-treatment of tissues with plant extract caused rightward non-parallel shift of PE (α-adrenoceptor agonist)-curves with suppression of maximum stimulatory effect, characteristic of a Ca^++^ antagonist like verapamil. In contrast, phentolamine, a competitive α-adrenergic receptors antagonist [31] caused a rightward parallel shift without suppression of maximum response as expected, thus ruling out the possibility of adrenoceptor blocking effect of extract. The endothelium-intact aorta profile helped in determining that the vasodilator effect of plant extract was independent of the endothelium, as was evident from the fact that the vasorelaxant effect of Gf.Cr in the endothelium-intact rat aorta was not antagonized by either L-NAME, a standard nitric oxide (NO) synthase inhibitor [32], atropine, an antagonist of ACh, that also brings about its effect by the release of NO [33] and indomethacin, a cyclooxygenase inhibitor [34], an enzyme responsible for the synthesis of prostacyclin (PGI_2_). The vascular endothelium plays a pivotal role in modulating vascular tone through release of mediators like NO and PGI_2_ which diffuses to the cells in the vicinity to cause relaxation [35,36]. The claim that the vasodilator effect was endothelium-independent is also strengthened from the aforementioned scenario, that when rat aorta was denuded of endothelium and the extract mediated relaxation of PE-induced contractions at similar concentration, as in intact preparations. Preliminary phytochemical analysis of *Gentiana floribunda* extract showed the presence of flavonoids, saponins, sterols, tannins and terpenes. Flavonoids are reported to possess hypotensive and vasodilator action via Ca^++^ antagonism [37,38] and the presence of such compounds in *Gentiana floribunda* may contribute in its observed effects. However the role of other constituents cannot be ignored. In acute toxicity testing, the plant extract was found safe up to maximum dose (3 g/kg) tested, which is in accordance with its wide therapeutic use.

## Conclusion

This study showed that *Gentiana floribunda* exhibits BP-lowering and vasodilatory effects mediated through inhibition of Ca^++^ influx via membranous calcium channels and its release from the intracellular stores and thus explains its folkloric repute as antihypertensive agent.

## Competing interests

The authors declare that they have no competing interest.

## Author’s contributions

AK carried out experimental work, data collection and evaluation, literature search and manuscript preparation. AUK identified the plant and helped in extraction. MRM and DDM raised funding, co-supervised research work and refined the manuscript for publication. All authors read and approved the final manuscript for publication.

## Pre-publication history

The pre-publication history for this paper can be accessed here:

http://www.biomedcentral.com/1472-6882/12/121/prepub
